# Isotopic Evidence for Early Trade in Animals between Old Kingdom Egypt and Canaan

**DOI:** 10.1371/journal.pone.0157650

**Published:** 2016-06-20

**Authors:** Elizabeth R. Arnold, Gideon Hartman, Haskel J. Greenfield, Itzhaq Shai, Lindsay E. Babcock, Aren M. Maeir

**Affiliations:** 1 Grand Valley State University, Department of Anthropology, Allendale, Michigan, United States of America; 2 University of Connecticut, Department of Anthropology, Center for Environmental Sciences and Engineering, Storrs, Connecticut, United States of America; 3 Department of Anthropology and St. Paul’s College, University of Manitoba, Winnipeg, Manitoba, Canada; 4 Ariel University, Israel Heritage Department and the Department of Land of Israel Studies and Archaeology, Ariel, Israel; 5 Bar-Ilan University, Department of Land of Israel Studies and Archaeology, Ramat Gan, Israel; Chinese Academy of Sciences, CHINA

## Abstract

Isotope data from a sacrificial ass and several ovicaprines (sheep/goat) from Early Bronze Age household deposits at Tell es-Safi/Gath, Israel provide direct evidence for the movement of domestic draught/draft and husbandry animals between Old Kingdom Egypt (during the time of the Pyramids) and Early Bronze Age III Canaan (ca. 2900–2500 BCE). Vacillating, bi-directional connections between Egypt and Canaan are known throughout the Early Bronze Age, but here we provide the first concrete evidence of early trade in animals from Egypt to Canaan.

## Introduction

Trade and cultural connections between Egypt and ancient Canaan (= the southern Levant) are known from many periods, commencing with the Chalcolithic period [1]. Trade in animals themselves is only attested from later periods (Middle Kingdom, Dynasty 12) [2], and in most cases, from Canaan to Egypt. Until recently, the accepted view was that intensive connections between Egypt and the Levant existed during the preceding EB I and EB II periods (though of fluctuating nature and mechanisms), while during the EB III, direct trade between Egypt and Canaan ceased or at least was substantially curtailed [3–8]. Archaeological evidence for these connections include both mundane (such as various agricultural products, fish from Egypt and oil from Canaan) and prestige objects (such as stone vessels and decorated stone palettes from Egypt, decorated pottery from Canaan) [3–7,9]. In light of new excavations and the new chronology for the EB southern Levant [10] (S1 Table), the supposed lack of connections during the EB III is being reassessed [11]. Given these new data, there is no doubt that exchange relationships continued, albeit on a much smaller scale, during the EB III.

**Fig 1:**
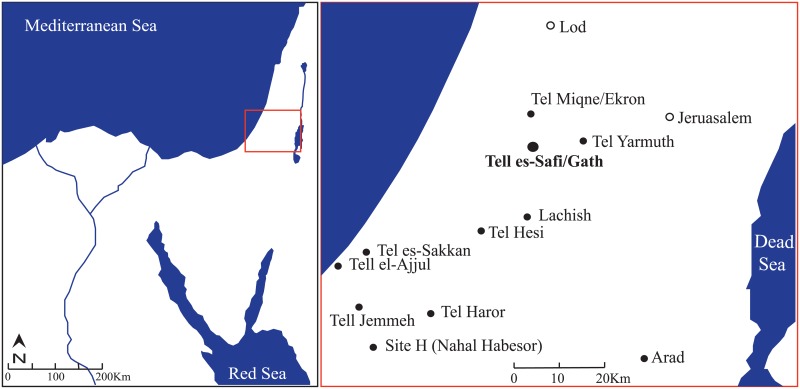
Location of Tell es-Safi/Gath in relation to selected Early Bronze Sites in the southern Levant (Canaan).

Several points can be noted for the trade patterns of early urban centers in the Levant. Most perishable subsistence items probably originated within a day or two walk of urban centers [7], a pattern that has long been observed for subsistence farmers—e.g. [12]. But the finished goods or their raw materials may come from much farther away. While the intensification of long distance trade is characteristic of early complex societies, such trade in raw materials and finished goods long pre-dates the emergence of EB urban centers in the region—e.g. [13,14]. In the EB, the scale of such exchange systems dramatically intensifies in order to satisfy the demands of growing urban populations and new elites. It can neither be demonstrated to be a cause nor an effect of the emergence of urban complex societies in the EB of the southern Levant since it seems to develop concomitantly [15]. The domestication of the donkey [16,17], which occurred sometime in the late 5^th^/early 4^th^ millennium BCE, could be seen as one of the factors in the amplification of trade, as it improved the ability to transport goods over a long distance—e.g. [14,18,19]. Evidence that donkeys were an important part of the EB III economy is seen at Tell es-Safi/Gath, Israel [20–23] (Fig 1), where the remains of several donkeys (and donkey-related objects) were found [11,24].

## The Zooarchaeological Sample

As previously discussed [24], the ass found at Tell es-Safi/Gath clearly represents a sacrificial deposit. All three molar teeth from the right mandible were sampled for isotopic analyses, i.e. carbon, oxygen and strontium. For comparative purposes and to develop a more complete picture of domestic animal management at the site, five ovicaprine teeth (four goats and one sheep) were also sampled from the same EB levels (S2 Table).

## Materials and Methods

All necessary permits were obtained for the described study, which complied with all relevant regulations. Excavation permits were issued to Dr. Maeir by the Israel Antiquities Authority (Permit # G-47/2004, G-65/2005, G-35/2006, G-54/2007, G-56/2008). Permits for exportation of material from Israel were issued to Dr. Greenfield by the Israel Antiquities Authority (Permit # 13840—July 25, 2012). Israel Nature and National Parks Protection Authority 2010/37996 issued to Dr. Hartman (for plant sampling in the Judean Hills, between 11/23/2010–11/22/2011). However, all of the plant sampling locations for this project were restricted to public lands that is not included in nature protected area for which explicit plant sampling permit is required. Collected species are not listed in the protected or endangered species list [25]. The amount of material collected above ground was 50 grams per plant. This quantity that does not harm shrubs and trees; same quantity that was collected from grassy species postdate growing season and included only dead plant parts.

### Modern reference collection and processing

Ten modern plant samples collected in the vicinity of the Tell es-Safi/Gath (S3 Table) were collected as modern base lines for local isotopic variability. Hartman and Richards [26] demonstrate that when there is high correlation between plant and invertebrate ^87^Sr/^86^Sr ratios, as there is in the Levant, it is suitable to use plants as a baseline for mapping of the modern bioavailable strontium distributions. Plant samples were prepared for strontium analysis with the methods as outlined by Copeland et al. [27].

Most of the region surrounding Tell es-Safi/Gath is sandwiched between Cretaceous–Eocene marine bedrock and Holocene alluvium, with bedrock ^87^Sr/^86^ Sr ratios ranging between 0.7078–0.7091). However, all of the outcrops excluding the alluvium will likely provide bioavailable ^87^Sr/^86^ Sr ratios ranging between ~0.7085–0.7091, depending on the degree of atmospheric deposition. The sampling strategy focused on the distinct geological outcrops that surround the site including the chalky bedrock from the Plio-Pleistocene calcareous sandstone and Holocene alluvium.

### Treatment of the archaeological sample—domestic animal teeth

Sequential sampling of teeth followed methods outlined by Bocherens et al. [28]. A series of 1.0 mm bands were drilled sequentially along the mesial lobe of each molar tooth [28]. Dentin and cementum were removed from the equine teeth [29] prior to sampling. Pretreatment of samples follows the methods described by Balasse [30].

For carbon and oxygen isotopic analyses, ~700 μg of the prepared sample were weighed into individual vessels and reacted with 100% phosphoric acid (H_3_PO_4_) at 70°C in an automated Kiel III carbonate device in which CO_2_ is liberated from enamel, cryogenically distilled, and subsequently flow to a Finnigan MAT 252 isotopic ratio mass spectrometer at the Anthropology Department Stable Isotope Laboratory and Mass Spectrometry Laboratory at the University of Illinois Urbana-Champaign. Analytical precision is typically ±0.07‰ for δ^13^C and ±0.14‰ for δ^18^O.

## Results

### Mapping local bioavailable ^87^Sr/^86^Sr ratios

**Fig 2:**
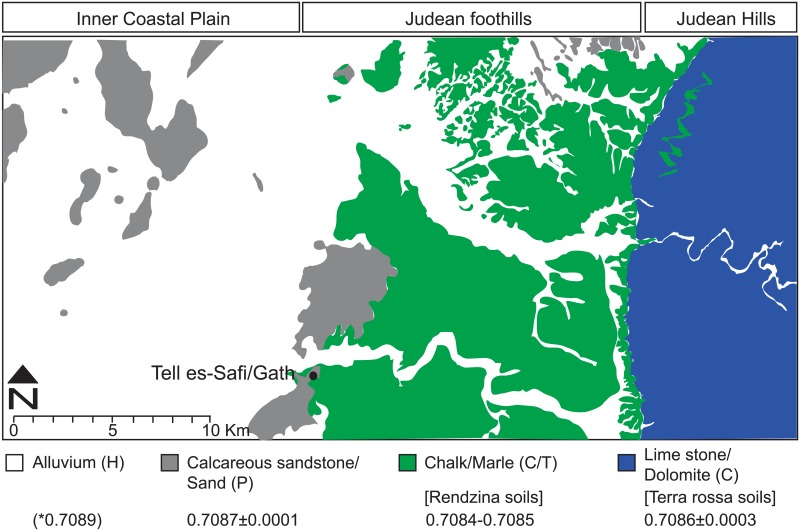
Map showing the bioavailable ^87^Sr/^86^Sr ratio isoscape of the Tell es-Safi/Gath region, based on local geological maps [34, 35]. For explanations, see methods section and S3 Table. *Alluvium strontium ratios vary depending on parent bedrock weathering and atmospheric deposition.

Local sampling of plants from different soil and bedrock types provides verification to bioavailable ^87^Sr/^86^Sr ratios [26]. It is clear that plants growing on rendzina soils that form on soft marl and chalk marine sedimentary bedrock (Fig 2) require calibration because of the contribution of atmospheric strontium. Atmospheric contribution relative to bedrock weathering varies positively with mean annual precipitation (MAP) [26]. High resolution cave speleothem isotope data provide credible evidence for wetter conditions during the EB III relative to present conditions by up to 150mm/yr [31]. Using the following Sr equation (Eq 1):

S87r/S86rrendzina=2 × 10−6×MAP+S87r/S86rbedrock−0.0005 (1)

cf. [26].

Setting MAP (Mean Annual Precipitation) to 600mm/yr (modern precipitation record is ~450mm/yr), with Paleocene and Eocene ^87^Sr/^86^ Sr _bedrock_ of 0.7077–0.7078, the resulting ^87^Sr/^86^ Sr _rendzina_ range is 0.7084–0.7085 [32,33], which characterizes the area to the east of the site.

While plant sampling took place on Holocene alluvium soils (vertisols), this sampling cannot provide a credible estimate for all alluvial soils due to their heterogeneous sources. *Terra rossa* (FAO: Cambisols and Phaeozems) soils develop on hard marine sedimentary bedrock (limestone and dolomite) mostly through atmospheric deposition [26,34]. This is evident from examination of plants growing on these soils since they incorporate bioavailable strontium with ^87^Sr/^86^Sr ratios of 0.7086±0.0003 (S3 Table) and [26]. These resemble modern rain water ratios measured in the southern Levant [34].

The bioavailable ^87^Sr/^86^Sr ratios are mapped into an isoscape (1:50,000 scale) providing estimated and measured bioavailable ^87^Sr/^86^Sr ratios for lithologies and soils, as described in Sneh [34,35]. By mapping the bioavailable ^87^Sr/^86^Sr ratios isoscape (1:50,000 scale), which is presented in the current study, it is possible to have a credible estimate of the geographic origin for local animal herds during the EB III. It also becomes possible to begin reconstructing herd management and movement patterns.

### Faunal carbon and oxygen

Carbon isotope values suggest that the ass (and its mother) was eating vegetation from a mixed C_3_/C_4_ environment with a high proportion of C_4_ vegetation in its early years, as reflected by the isotope values of M_1_ and M_2_ (S4 Table; S1 Fig). Subsequently, the animal moved into and grazed in an area with a greater proportion of C_3_ vegetation, as reflected in the more negative *δ*^13^C values in the third molar. From these values, it is clear that the ass was not born and raised locally at Tell es-Safi/Gath, but was imported and lived within the local environment surrounding the site only for a short period of time before its death. The ovicaprine data (S5 Table and S1 Fig) suggest a very different pattern—i.e. consumption of local southern Levantine C_3_ vegetation. The only exception is from OC#3 (a goat) that plots in the same range with the ass suggesting that it too was raised outside the local region.

**Fig 3:**
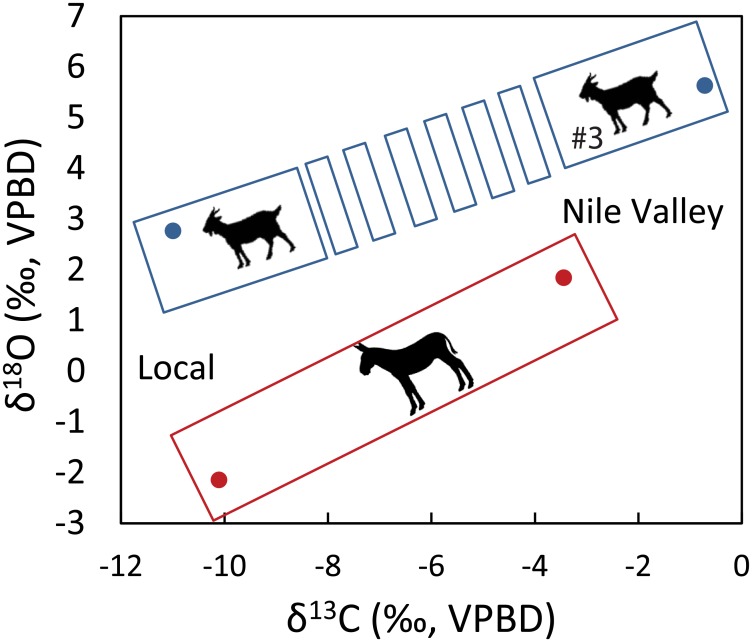
Local vs. Nile valley bivariate plotting of mean intra (ass; red frame) and inter-tooth (ovicaprine; dashed blue frame) δ^18^O and δ^13^C values.

The “foreign” plotting OC#3 (S1 Fig) shows a positive shift in δ^18^O values relative to the “local” ovicaprines (Δ^18^O _local—foreign ovicaprine_ = 2.87‰). A similar shift in δ^18^O values is measured between the mean of the sacrificial ass M_1_+M_2_ (δ^18^O = 1.84‰) and the most negative M_3_ (δ^18^O = -2.15‰) molars (Δ^18^O _M1 +M2 –M3 ass_ = 3.99‰) (Fig 3). The sacrificial ass “foreign” δ^18^O values fully resemble archaeological human tooth and bone carbonate values that were analyzed at different locations along the Nile valley (+1.05±1.02‰) [36]. While the ass δ^18^O values are more negative than those measured in the ovicaprines, this difference is the result of physiological differences between large and small/medium sized herbivores [37].

## Discussion

Three independent isotopic proxies provide unique environmental and spatial evidence that separate local from imported livestock. Furthermore, evaluation of the early life history of the sacrificial ass and one of the ovicaprines (OC#3) suggest that they originated in the Nile Valley (Fig 3).

Carbon isotopes (δ^13^C) measured for modern Levantine herbivores reflect the dominance of C_3_ vegetation in Mediterranean climate [38]. The large range of values measured in local C_3_ vegetation (δ^13^C site means: -29 –(-25) ‰) reflects different plant life in response to seasonal and annual availability of water [39,40]. While C_4_ grasses are part of the Mediterranean flora [41], their abundance and specifically those of C_4_ chenopods (~-10 –(-14)‰) increases in steppe and desert environments [42]. Even in the Negev and Sinai deserts to the south, ethnobotanical study of husbandry subsistence on local plants show great preference of donkeys for the consumption of aromatic (etheric) C_3_ plant species [43]. This observation is supported by a study of Negev and Sinai desert wild bovids (*Capra ibex nubiana*, *Gazella dorcas*), who show only secondary contribution of C_4_ vegetation into a C_3_ dominated diet [44]. The nearest geographic location where C_4_ vegetation can serve as primary fodder for herbivores is the Nile River delta and the riparian banks of the Nile River where tropical C_4_ sedges prevail [45,46]. As such, the primary indicator for Nile Valley origins is the dominance of C_4_ vegetation (>50%) [47], as indicated by the δ^13^C carbonate values measured in OC#3 and early tooth formation of the ass (M_1_+M_2_) (S1 Fig). To reach the δ^13^C values measured in the ass and OC#3 teeth, C_4_ vegetation should make up a major part of available plant biomass. While locations along the Red Sea coast show high proportion of C_4_ plant species [42], they do not form the majority of edible plant biomass. This contrasts with the δ^13^C values measured in local ovicaprines that show a primary subsistence on C_3_ vegetation. The nearest environmental location where C_4_ vegetation is also associated with biomass productivity that can support large livestock populations is the Nile River valley and delta, where C_4_ sedges (Cyperaceae; *Cyperus papyrus*) as well as commercial C_4_ crops make good fodder for husbandry animals. Bernhardt *et al*. [45] document pollen evidence for Cyperaceae abundance in the Nile River delta during the EB and δ^13^C values measured in ancient Egyptian husbandry animals reveals a common contribution of C_4_ component in their diets [46].

Tooth enamel carbonate δ^18^O values provide a similar conclusion. The mean δ^18^O values of rainfall measured today in the coastal area of the southern Levant in proximity to Tell es-Safi/Gath varies between -3.5– (-5)‰ (VSMOW). In warmer and drier desert locations such as the Arava valley, the mean value is -1.8‰ and even higher in ephemeral surface water desert locations [49,50]. Surface water (springs) in eastern Sinai can also show similarly evaporative values, though the majority of reported values resemble Mediterranean coastal ranges [51]. Finally, in Egypt, the Nile River water (~3.8‰ near Cairo) is the primary source of water for this hyper arid region, and reflects evaporative environmental conditions throughout the long Nile River course [52].

**Fig 4:**
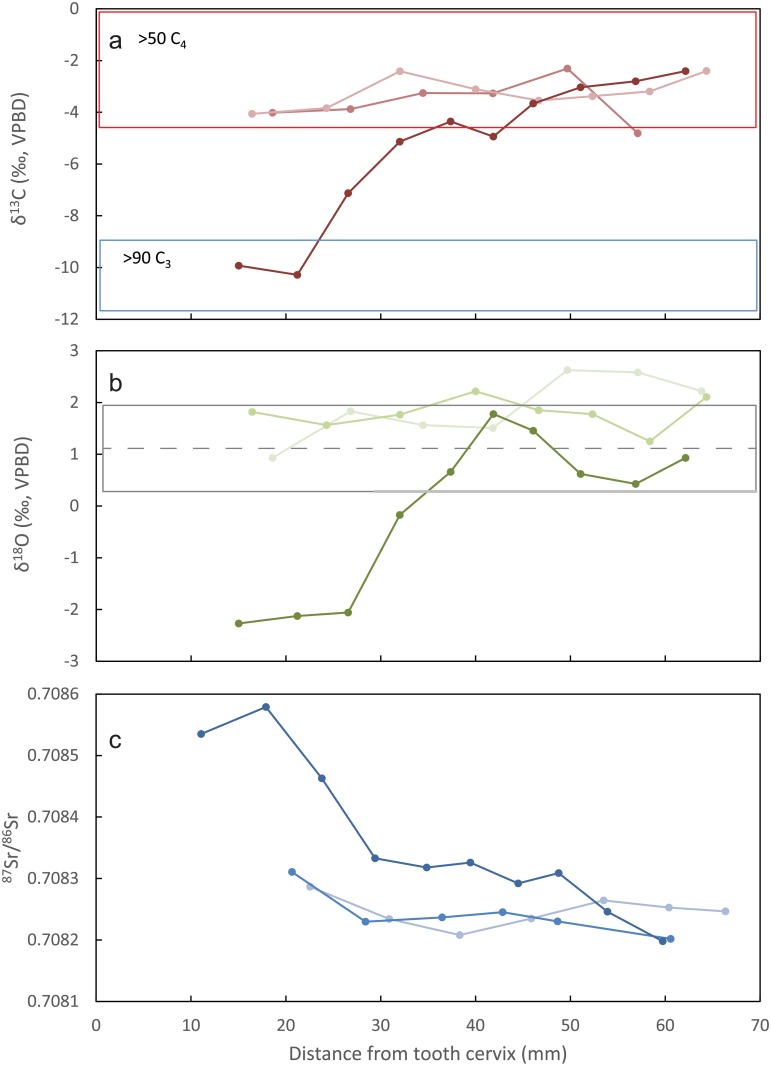
Sequential isotopic carbon [a], oxygen [b], strontium [c] sampling of sacrificial ass mandibular teeth. b—The dashed red line represents the mean oxygen isotope values of Egyptian archaeological humans. The solid redline is *+* 1 stdev [48].

The early life stage of the ass (M_1_+M_2_) includes a uniform seasonal pattern (+1.84±0.46‰) that indicates a permanent and relatively unchanging water source. Furthermore, the early life δ^18^O values fall in the range of values measured in human burials excavated along the Nile Valley (+1.05±1.0‰) [36]. While humans and equids differ in body mass and metabolism, both are large enough to reflect the dominant local environmental water conditions [37]. Once moved to Tell es-Safi/Gath, a rapid negative shift of 4‰ is recorded in the ass’s third molar (-2.15±0.18‰) (Fig 4). A similar difference exists in the measurements between local ovicaprines (OC #1, 2, 4, and 5) and the “exotic” OC #3 (that we propose to have been raised in the Nile Valley). Ovicaprines, as medium sized ruminants, derive much more of their body water from evaporative sources such as their diet and metabolic water production. This accounts for the consistently higher δ^18^O values relative to the ass [37,53]. Year round supply of water in the hyper-arid deserts of Sinai depends on springs fed by water with isotopic compositions that resemble those measured in the coastal region of the southern Levant [51]. As such, this excludes again the possibility that the ass and OC #3 arrived from an alternative desert location.

Using the isoscape map and defined ^87^Sr/^86^Sr ratios for the local region, it becomes evident that the bulk of the ovicaprine sample represents evidence for local grazing on rendzina soils that develop on Tertiary soft marine sedimentary bedrock. These cover an expansive region that borders Tell es-Safi/Gath from the east (S6 Table). This hilly region is very suitable for the herding of both grazers (sheep) and browsers (goats), while the flatter dark brown alluvium vertisol soils located west of the site that extend to the seashore are more suitable for rain-based cereal agriculture (Fig 2) [32]. The exception is sample OC #3 that plots in the same range with the ass. Both OC #3 and early life stage of the ass (M_1_ + M_2_) provide mean ^87^Sr/^86^Sr ratios that are foreign to the Tell es-Safi/Gath region (0.7080 and 0.7082 respectively–S7 Table).

The isotopic results from Tell es-Safi/Gath indicated that the Nile Valley of Egypt is the origin of the sacrificial ass and OC #3. Faunal remains from the Nile Valley provide a broad range of ^87^Sr/^86^Sr ratios that reflect mixing of heterogeneous sources [54]. On the other hand, human burials from the Nile Valley yield tighter ratio ranges to which the ass and OC#3 can be related (Memphis in Lower Egypt, $\overset{-}{x}\;=\;0.7078\pm 0.003$; range 0.7074–0.70870) [54]. Although not exclusive to the Nile Valley, these ratios do not contradict the other two independent lines of isotopic evidence—carbon and oxygen. The higher radiogenic ^87^Sr/^86^Sr profile of the sacrificial ass in its final stages of tooth formation (M_3_), when it is brought to Tell es-Safi/Gath (0.7086; Fig 4c), plots closer to plant fodder that would have been raised in the vicinity of the site on the deep alluvium agricultural fields to the west of the site (Fig 2).

## Conclusions

These data provide direct evidence for movement and trade of domestic draught/draft and husbandry animals between Old Kingdom Egypt and Canaan during the EB III, and provide insight into the character of the connections between the two regions during this period. The sacrificial ass formed its early tooth development in the Nile Valley and later migrated to Tell es-Safi/Gath where it completed the formation of its third molar. This corroborates textual and other archaeological information that already pointed toward the existence of long distance trade, apparently in donkey caravans, between Egypt and Canaan during this early urban period.

The data which we present suggests several additional aspects to what was previously assumed. While the presence of a Nilotic draft animal is conceivable given the already existing knowledge that commodities and exotic goods were transported in caravans between the Nile Valley and Fertile Crescent, the finding of a Nile Valley goat (OC #3) is completely novel as it suggests trade in commodities (domestic animals) whose origins were previously invisible to archaeologists. This includes domestic animals for consumption (i.e. ovicaprines) and the very asses used in the transportation of goods. While trade in animals from Canaan to Egypt are known from later periods [2,55], there is little information on the trade of animals in the opposite direction—and in particular during the Egyptian Old Kingdom. And in fact, it was often assumed that the majority of trade goods sent from Egypt to Canaan were of types unavailable in the Levant (such as prestige objects and specific foodstuffs) [1]. Here we provide evidence that there were commodities previously not thought to have been among those traded between Egypt and the Levant (animals) were in fact part of these trade connections. These results imply that the character of trade relations between Egypt and the Levant during the EB III may have been broader and more multi-faceted than has been previously assumed—e.g. [1,3–5,7].

Finally, it can be noted that while Tel Yarmuth, a neighboring EB polity of Tell es-Safi/Gath, has been suggested as the primary polity in the region during the EB III [56,57], this and other data from Tell es-Safi/Gath force a reconsideration of the nature of such relationships [11,58]. As the isotope data point to a vibrant trade connection with Egypt, this may hint to the important status and supra-regional role of Tell es-Safi/Gath during this period. Being extremely large in size (c. 24 ha), surrounded by a major fortification system [59], and closer to the major coastal trade and transport routes than Tel Yarmuth, Tell es-Safi/Gath may have controlled some of the trade connections to the more interior settlements in its hinterland (such as Tel Yarmuth). Hence, the relationship between these two, and other, early urban centers in the region should be reevaluated, since they are likely more of competing peer-polities or city-states in nature.

## Supporting Information

S1 Fig Bivariate plots of individual ovicaprines (digits 1–5, blue dots) and sacrificed ass (M_1_-M_3_, red dots) tooth enamel δ^13^C values against δ^18^O values (a), and ^87^Sr/^86^Sr ratios (b). Error bars represent ±1σ.
(EPS)

S1 Table Phases of the Early Bronze Age in Israel. (DOCX)

S2 Table Contextual and excavation data of sacrificial ass (*Equus asinus*) and ovicaprines. (DOCX)

S3 Table Local baseline modern plants. (DOCX)

S4 Table Carbon and oxygen isotope values for sacrificial ass (*Equus asinus*). (DOCX)

S5 Table Carbon and oxygen isotope values for ovicaprines. (DOCX)

S6 Table ^87^Sr/^86^Sr ratios for ovicaprines. (DOCX)

S7 Table ^87^Sr/^86^Sr for the sacrificial ass (*Equus asinus*). (DOCX)
