# Supplementary material for: Isotopic Evidence for Early Trade in Animals between Old Kingdom Egypt and Canaan
Source: PLoS One. 2016 Jun 20;11(6):e0157650. doi: 10.1371/journal.pone.0157650 (PMC4913912; doi:10.1371/journal.pone.0157650)
Supplement: S1 Table — (DOCX) [file pone.0157650.s002.docx]

| **S1 Table. Phases of the Early Bronze Age in Israel.** | | |  |
| --- | --- | --- | --- |
| **Period** | **Abbreviation** | **Chronology** | **Characteristics** |
| Early Bronze I | EBA I | 3500-3000 BCE | Proto-urban |
| Early Bronze II | EBA II | 3000-2600 BCE | Urban |
| Early Bronze III | EBA III | 2600-2300 BCE | Urban |
| Early Bronze IV | EB IV | 2300-2000 BCE | Collapse |
