# Supplementary material for: Isotopic Evidence for Early Trade in Animals between Old Kingdom Egypt and Canaan
Source: PLoS One. 2016 Jun 20;11(6):e0157650. doi: 10.1371/journal.pone.0157650 (PMC4913912; doi:10.1371/journal.pone.0157650)
Supplement: S2 Table — (DOCX) [file pone.0157650.s003.docx]

| **S2 Table. Contextual and excavation data of sacrificial ass and ovicaprines** | | | | |  |  |  |
| --- | --- | --- | --- | --- | --- | --- | --- |
|  |  | |  |  |  |  |  |
| **Sample ID** | **OC 1** | **OC 2** | **OC 3** | **OC 4** | **OC 5** | **Sacrificial ass** |  |
| **Site** | Tell es-Safi | Tell es-Safi | Tell es-Safi | Tell es-Safi | Tell es-Safi | Tell es-Safi |  |
| **Area** | E | E | E | E | E | E |  |
| **Year excavated** | 2004 | 2005 | 2006 | 2008 | 2007 | 2008 |  |
| **Locus** | 74505 | 745 | 94605 | 114602 | 104308 | 114506 |  |
| **Basket** | 745082 | 845027 | 946015 | 1146004 | 1043038 | 1145054 |  |
| **Tooth** | Lower M3 | Lower M1 | Lower M1 | Lower M1 | Lower M2 | Lower M1-M3 |  |
| **Species** | *Capra hircus* | *Capra hircus* | *Capra hircus* | *Ovis aries* | *Capra hircus* | *Equus asinus* |  |
| **Curation location** | UofManitoba | UofManitoba | UofManitoba | UofManitoba | UofManitoba | UofManitoba |  |
