# Supplementary material for: Isotopic Evidence for Early Trade in Animals between Old Kingdom Egypt and Canaan
Source: PLoS One. 2016 Jun 20;11(6):e0157650. doi: 10.1371/journal.pone.0157650 (PMC4913912; doi:10.1371/journal.pone.0157650)
Supplement: S3 Table — (DOCX) [file pone.0157650.s004.docx]

| **S3 Table. Local baseline modern plants** | | |  |  |
| --- | --- | --- | --- | --- |
| **Sample #** | **Soil Type** | **Name** | **Plant Characteristic** | **^87^Sr/^86^Sr** |
| MB 1 | Kurkar | Unidentified grass | Grass | 0.708810 |
| MB 2 | Kurkar | *Asphodelius ramosus* | Non-ligneous | 0.708810 |
| MB 3 | Alluvium vertisol | *Eucalyptus camaldulensis (Dehnh.)* | Ligneous | 0.708865 |
| MB 4 | Terra rossa | *Quercus calliprinos* | Ligneous | 0.708592 |
| MB 5 | Terra rossa | *Pistacia lentiscus* | Ligneous | 0.708717 |
| MB 6 | Kurkar | *Ceratonia siliqua L.* | Tree | 0.708631 |
| MB 7 | Terra rossa | *Erucaria hispanica (L) Druce* | Non-ligneous | 0.708720 |
| MB 8 | Alluvium | *Sinapis arvensis (L)* | Non-ligneous | 0.708865 |
| MB 9 | Terra rossa | *Stipa capensis (Thunb)* | Non-ligneous | 0.708637 |
| MB 10 | Kurkar | *Hyparrhenia hirta* (C_4_ Grass) | Non-ligneous | 0.708675 |
