# Supplementary material for: Isotopic Evidence for Early Trade in Animals between Old Kingdom Egypt and Canaan
Source: PLoS One. 2016 Jun 20;11(6):e0157650. doi: 10.1371/journal.pone.0157650 (PMC4913912; doi:10.1371/journal.pone.0157650)
Supplement: S4 Table — (DOCX) [file pone.0157650.s005.docx]

| **S4 Table. Carbon and oxygen isotope values for sacrificial ass (*Equus asinus*)** | | | | |  |
| --- | --- | --- | --- | --- | --- |
| **Sample code** | **Distance from enamel/root junction (mm)** | **Tooth** | **δ^13^CvPDB** | **δ^18^OvSMOW** | **δ18OvPDB** |
| TS01 | 18.60 | M1 | 4.0 | 31.82 | 0.93 |
| TS03 | 26.81 | M1 | -3.9 | 32.75 | 1.83 |
| TS05 | 34.46 | M1 | -3.3 | 32.48 | 1.56 |
| TS07 | 41.82 | M1 | -3.3 | 32.42 | 1.51 |
| TS09 | 49.67 | M1 | -2.3 | 33.57 | 2.62 |
| TS11 | 57.10 | M1 | -4.8 | 33.53 | 2.58 |
| TS13 | 63.82 | M1 | -4.2 | 33.15 | 2.22 |
| TS15 | 16.44 | M2 | -4.1 | 32.74 | 1.82 |
| TS17 | 24.29 | M2 | -3.8 | 29.25 | -1.56 |
| TS19 | 32.03 | M2 | -2.4 | 32.68 | 1.77 |
| TS21 | 40.00 | M2 | -3.1 | 33.15 | 2.22 |
| TS23 | 46.64 | M2 | -3.5 | 32.77 | 1.85 |
| TS25 | 52.33 | M2 | -3.4 | 32.69 | 1.78 |
| TS27 | 58.36 | M2 | -3.2 | 32.15 | 1.25 |
| TS29 | 64.35 | M2 | -2.4 | 33.04 | 2.11 |
| TS31 | 15.03 | M3 | -9.9 | 28.52 | -2.27 |
| TS33 | 21.21 | M3 | -10.3 | 28.67 | -2.12 |
| TS35 | 26.56 | M3 | -7.1 | 28.74 | -2.06 |
| TS37 | 32.01 | M3 | -5.1 | 30.69 | -0.17 |
| TS39 | 37.37 | M3 | -4.4 | 31.54 | 0.66 |
| TS41 | 41.87 | M3 | -4.9 | 32.70 | 1.78 |
| TS43 | 46.08 | M3 | -3.7 | 32.36 | 1.46 |
| TS45 | 51.06 | M3 | -3.0 | 31.50 | 0.62 |
| TS47 | 56.88 | M3 | -2.8 | 31.30 | 0.43 |
| TS49 | 62.13 | M3 | -2.4 | 31.82 | 0.93 |
