# Supplementary material for: Isotopic Evidence for Early Trade in Animals between Old Kingdom Egypt and Canaan
Source: PLoS One. 2016 Jun 20;11(6):e0157650. doi: 10.1371/journal.pone.0157650 (PMC4913912; doi:10.1371/journal.pone.0157650)
Supplement: S5 Table — (DOCX) [file pone.0157650.s006.docx]

**S5 Table. Carbon and oxygen isotope values for ovicaprines**

| **Individual** | **Sample code** | **Tooth** | **Distance from enamel/root junction (mm)** | **δ^13^CvPDB** | **δ^18^OvSMOW** | **δ^18^OvPDB** |
| --- | --- | --- | --- | --- | --- | --- |
| OC#5 | LB 05 | M2 | 14.02 | -10.58 | 33.27 | 2.34 |
| OC#1 | LB 07 | M3 | 4.34 | -9.4 | 33.93 | 2.97 |
| OC#1 | LB 11 | M3 | 9.30 | -11.6 | 33.61 | 2.66 |
| OC#1 | LB 13 | M3 | 11.56 | -11.1 | 34.05 | 3.09 |
| OC#1 | LB 15 | M3 | 14.44 | -9.2 | 32.58 | 1.67 |
| OC#1 | LB 17 | M3 | 17.34 | -8.2 | 31.80 | 0.91 |
| OC#1 | LB 19 | M3 | 20.20 | -7.4 | 31.65 | 0.76 |
| OC#2 | LB 21 | M1 | 11.18 | -11.0 | 36.46 | 5.43 |
| OC#2 | LB 23 | M1 | 14.36 | -10.8 | 36.49 | 5.46 |
| OC#2 | LB 25 | M1 | 17.00 | -10.9 | 35.80 | 4.79 |
| OC#2 | LB 27 | M1 | 19.60 | -11.0 | 35.28 | 4.28 |
| OC#2 | LB 29 | M1 | 22.34 | -10.7 | 34.36 | 3.39 |
| OC#2 | LB 31 | M1 | 25.60 | -11.0 | 32.42 | 1.51 |
| OC#3 | LB 33 | M1 | 5.56 | 1.8 | 35.10 | 4.11 |
| OC#3 | LB 35 | M1 | 8.54 | 2.1 | 36.37 | 5.34 |
| OC#3 | LB 37 | M1 | 11.90 | 0.3 | 37.51 | 6.45 |
| OC#3 | LB 39 | M1 | 14.68 | -2.4 | 37.37 | 6.31 |
| OC#3 | LB 41 | M1 | 17.54 | -5.4 | 36.99 | 5.95 |
| OC#4 | LB 43 | M1 | 16.62 | -12.0 | 33.96 | 3.00 |
| OC#4 | LB 45 | M1 | 20.20 | -12.9 | 33.93 | 2.97 |
| OC#4 | LB 47 | M1 | 23.04 | -14.2 | 32.62 | 1.70 |
