# Supplementary material for: Isotopic Evidence for Early Trade in Animals between Old Kingdom Egypt and Canaan
Source: PLoS One. 2016 Jun 20;11(6):e0157650. doi: 10.1371/journal.pone.0157650 (PMC4913912; doi:10.1371/journal.pone.0157650)
Supplement: S6 Table — (DOCX) [file pone.0157650.s007.docx]

| **S6 Table. ^87^Sr/^86^Sr ratios for ovicaprines.** | | | |  |
| --- | --- | --- | --- | --- |
| **Individual** | **Sample code** | **Tooth** | **Distance from enamel/root junction (mm)** | **87Sr/86Sr** |
| OC#1 | LB08 | M3 | 5.58 | 0.708773 |
| OC#1 | LB10 | M3 | 8.24 | 0.70899 |
| OC#1 | LB12 | M3 | 10.28 | 0.708707 |
| OC#1 | LB14 | M3 | 13.00 | 0.708586 |
| OC#1 | LB16 | M3 | 15.64 | 0.708597 |
| OC#1 | LB18 | M3 | 18.68 | 0.708574 |
| OC#1 | LB20 | M3 | 21.46 | 0.708656 |
| OC#2 | LB22 | M1 | 12.76 | 0.708426 |
| OC#2 | LB24 | M1 | 15.72 | 0.708451 |
| OC#2 | LB26 | M1 | 18.24 | 0.708409 |
| OC#2 | LB28 | M1 | 21.28 | 0.708384 |
| OC#2 | LB30 | M1 | 23.60 | 0.708378 |
| OC#2 | LB32 | M1 | 26.60 | 0.708359 |
| OC#3 | LB34 | M1 | 7.14 | 0.708049 |
| OC#3 | LB36 | M1 | 9.70 | 0.708073 |
| OC#3 | LB38 | M1 | 13.38 | 0.708052 |
| OC#3 | LB40 | M1 | 15.80 | 0.708023 |
| OC#4 | LB42 | M1 | 15.06 | 0.708445 |
| OC#4 | LB44 | M1 | 18.18 | 0.708428 |
| OC#4 | LB46 | M1 | 21.48 | 0.70847 |
| OC#5 | LB04 | M2 | 12.48 | 0.708447 |
| OC#5 | LB06 | M2 | 15.12 | 0.708431 |
