# Supplementary material for: Isotopic Evidence for Early Trade in Animals between Old Kingdom Egypt and Canaan
Source: PLoS One. 2016 Jun 20;11(6):e0157650. doi: 10.1371/journal.pone.0157650 (PMC4913912; doi:10.1371/journal.pone.0157650)
Supplement: S7 Table — (DOCX) [file pone.0157650.s008.docx]

| **S7 Table. ^87^Sr/^86^Sr for the sacrificial ass (*Equus asinus*)** | | | |
| --- | --- | --- | --- |
| **Sample code** | **Tooth** | **Distance from enamel/root junction (mm)** | **^87^Sr/^86^Sr** |
| TS02 | M1 | 22.56 | 0.708287 |
| TS04 | M1 | 30.89 | 0.708234 |
| TS06 | M1 | 38.33 | 0.708208 |
| TS08 | M1 | 45.88 | 0.708235 |
| TS10 | M1 | 53.51 | 0.708265 |
| TS12 | M1 | 60.39 | 0.708253 |
| TS14 | M1 | 66.35 | 0.708247 |
| TS16 | M2 | 20.64 | 0.708311 |
| TS18 | M2 | 28.40 | 0.708230 |
| TS20 | M2 | 36.47 | 0.708237 |
| TS22 | M2 | 42.86 | 0.708245 |
| TS24 | M2 | 48.64 | 0.708230 |
| TS28 | M2 | 60.57 | 0.708202 |
| TS30 | M3 | 11.06 | 0.708535 |
| TS32 | M3 | 17.90 | 0.708579 |
| TS34 | M3 | 23.80 | 0.708463 |
| TS36 | M3 | 29.40 | 0.708333 |
| TS38 | M3 | 34.84 | 0.708318 |
| TS40 | M3 | 39.46 | 0.708326 |
| TS42 | M3 | 44.49 | 0.708292 |
| TS44 | M3 | 48.76 | 0.708309 |
| TS46 | M3 | 53.90 | 0.708246 |
| TS48 | M3 | 59.74 | 0.708198 |
